# Analysis of *Complementary Sex-Determiner* (*csd*) Allele Diversity in Different Honeybee Subspecies from Italy Based on NGS Data

**DOI:** 10.3390/genes13060991

**Published:** 2022-05-31

**Authors:** Gianluigi Paolillo, Maria Grazia De Iorio, Joel F. Soares Filipe, Federica Riva, Alessandra Stella, Gustavo Gandini, Giulio Pagnacco, Barbara Lazzari, Giulietta Minozzi

**Affiliations:** 1 Dipartimento di Medicina Veterinaria (DIMEVET), University of Milan, 26900 Lodi, Italy; gianluigi.paolillo@unimi.it (G.P.); mariagrazia.deiorio1993@gmail.com (M.G.D.I.); joel.soares@unimi.it (J.F.S.F.); federica.riva@unimi.it (F.R.); gustavo.gandini@unimi.it (G.G.); 2 IBBA-CNR, 20133 Milano, Italy; stella@ibba.cnr.it (A.S.); giulio.pagnacco@unimi.it (G.P.)

**Keywords:** *Apis mellifera*, *csd* alleles, hypervariable region, sex determination, biodiversity

## Abstract

Sexual regulation in *Apis mellifera* is controlled by the *complementary sex-determiner (csd)* gene: females (queens and workers) are heterozygous at this locus and males (drones) are hemizygous. When homozygous diploid drones develop, they are eaten by worker bees. High *csd* allelic diversity in honeybee populations is a priority for colony survival. The focus of this study is to investigate *csd* variability in the genomic sequence of the hypervariable region (HVR) of the *csd* gene in honeybee subspecies sampled in Italy. During the summer of 2017 and 2018, worker bees belonging to 125 colonies were sampled. The honeybees belonged to seven different *A. mellifera* subspecies: *A. m. ligustica*, *A. m. sicula*, *A. m cecropia*, *A. m. carnica*, *A. m. mellifera*, Buckfast and hybrid Carnica. Illumina genomic resequencing of all samples was performed and used for the characterization of global variability among colonies. In this work, a pipeline using existing resequencing data to explore the *csd* gene allelic variants present in the subspecies collection, based on *de novo* assembly of sequences falling within the HVR region, is described. On the whole, 138 allelic sequences were successfully reconstructed. Among these, 88 different alleles were identified, 68 of which match with *csd* alleles present in the NCBI GenBank database.

## 1. Introduction

Honeybees, like other hymenopteran insects, are haplodiploid: females (queen and workers) develop from fertilized diploid eggs, while males (drones) develop from unfertilized haploid eggs [1]. The *complementary sex-determiner* (*csd*) gene has been identified to be the primary gene involved in sexual regulation in honeybees [2]. Honeybees heterozygous at the *csd* locus develop into females, while haploid hemizygous bees develop as drones [3]. When diploid eggs are homozygous at the *csd* gene, diploid males are formed, but subsequently identified and eaten by worker bees [4]. The *csd* gene derives from its conserved paralog gene *feminizer* (*fem*) by gene duplication; *fem* regulates female development and is located 12 kb upstream of *csd* [5,6].

The *csd* gene is composed of nine exons divided into three clusters that encode an SR-type protein with a putative protein-binding function [2,7]. The third cluster (exons six to nine) contains a highly polymorphic region defined as the hypervariable region (HVR), which shows high variability and includes asparagine/tyrosine repeats varying significantly in number among alleles [2,8,9,10]. In 2013, Beye et al. determined that at least five amino acid differences and length variations between the two alleles of the same honeybee are sufficient to regularly induce the female pathway [8]. Initial findings estimated that the number of *csd* alleles in *Apis mellifera* populations ranged from 10 to 13 [11,12]. Subsequent studies have been conducted worldwide to identify the number of alleles circulating in the honeybee population, identifying a much higher number of alleles. 

In detail, Cho et al. identified 18 distinct alleles by 27 workers sampled from a single hive in Michigan [13]. In Slovenia (Litija) and South Africa (Pretoria), Hasselmann found 15 *csd* alleles from hundreds of embryos collected from two colonies in each location [14]. In New Zealand, Hyink et al. screened the *csd* locus by sampling six purple eye drone pupae from 42 hives, obtaining both allele sequences from 35 of the 42 queens, and only one allele sequence from the remaining seven. Based on these 77 amino acid sequences, they identified 16 different alleles, six of which were new alleles that had not been sequenced previously [15]. In 2019, Kaskinova et al. sampled 42 drones belonging to 15 different colonies in Russia and found 20 different alleles [16]. In 2020, Kolics et al. analyzed, with a new bioinformatic approach, 12 bee samples (4 workers, 4 drones and 4 queens) from three different subspecies (*A. m. carnica*, *A. m. ligustica* and *A. m. caucasica*) and 8 samples of honey collected in China, Hungary, Japan and Georgia. Among these samples, they found a total of 25 *csd* alleles [17]. In 2021, Bovo et al. analyzed DNA from honey samples from 12 different colonies and identified 160 different *csd* alleles [18].

However, some studies propose that the number of circulating alleles in the worldwide honeybee population is significantly underestimated. Wang et al. found a total of 79 haplotypes by analyzing 50 workers for each of the six different *Apis mellifera* subspecies they sampled (*A. m. anatolica*, *A. m. caucasica*, *A. m. carnica*, *A. m. carpatica*, *A. m. ssp*. and *A. m. ligustica*) [19]. Lechner et al., using a dataset of 244 *csd* sequences sampled worldwide, demonstrated that the total number of *csd* alleles in *A. mellifera* ranges from 53 locally to 87 worldwide [9]. In 2017, Zareba et al., in agreement with Wang and Lechner, identified 121 different alleles by analyzing 193 colonies in Poland [20]. 

A recent comprehensive study conducted on 652 sequences found in GenBank confirmed that the number of circulating alleles is very high. In this set, which represents a vast geographic area and eight subspecies, 225 alleles were found, grouping sequences by identity and numbering them consecutively from the most common to the least (Amelcsd-HVR1-Amelcsd-HVR225) [21]. 

The high number of csd alleles reflects the high honeybee genetic diversity due to adaptation to different global environmental conditions [22]. However, high attention must be given to the number of circulating alleles in managed breeding honeybee populations. 

Over the years, different approaches to determine *csd* alleles have been explored, usually based on Sanger sequencing. Currently, *csd* alleles of a diploid female honeybee are frequently determined through the analysis of *csd* sequences of six to eight male descendants by Sanger sequencing [15,19,20,23].

In 2020, Kolics et al. [17] used a high-throughput sequencing method to determine both *csd* alleles from honeybee queens and from honey samples. DNA was extracted from clipped wings of queen bees and worker bees, and locus targeted Illumina sequencing was performed, obtaining *csd* alleles directly from the diploid queens. In 2021, Bovo et al. [18] used a semiconductor-based sequencing technology (Ion Torrent) on honey samples to assess *csd* allelic variability from 12 different colonies. To further investigate the applicability of bioinformatic approaches for *csd* allele assessment and variability investigation from NGS data, we propose an alternative method based on local de novo assembly of Illumina reads matching the HVR, integrated by manual inspection and curation. The aim of this work was to exploit the existing Illumina genomic resequencing data collection described in Minozzi et al. [24] and retrieve *csd* HVR allele-specific sequences from diploid worker bees, thus describing the inter- and intra- genetic variability of seven *Apis mellifera* subspecies on 125 diploid worker bees sampled in different Italian regions.

## 2. Materials and Methods

### 2.1. Sampling

One hundred twenty-five worker colonies were sampled between summer 2017 and summer 2018 [24] in twelve different Italian regions: Abruzzo, Emilia Romagna, Liguria, Lombardy, Marche, Piedmont, Sicily, Trentino, Tuscany, Umbria and Veneto. Newly hatched worker bees were collected from each hive and used to perform a whole genome DNA sequencing analysis. Bees were individually placed in 1.5 mL Eppendorf tubes containing 95% ethanol and kept at −4 °C. The 125 colonies belonged to seven different subspecies [24], two autochthonous *A. m. ligustica* (*n* = 61) and *A. m. sicula* (*n* = 6); three allochthonous *A. m. mellifera* (*n* = 4), *A. m. carnica* (*n* = 8) and *A. m. cecropia* (*n* = 1); and two hybrids: *A. m. carnica* by *A. m. ligustica* hybrids (*n* = 2), and Buckfast bees (*n* = 43). 

### 2.2. DNA Extraction, Library Preparation, Sequence Processing and Alignment

All details of DNA extraction to sequence processing and alignment can be found in Minozzi et al. [24]. Based on the existing alignment, a pipeline was set up to perform local de novo assembly of sequences falling within the HVR region and determine the *csd* gene allelic variants present in the subspecies collection.

### 2.3. De Novo Assembly and Analysis of Sample-Specific HVR Allele Consensus Sequences

Samtools was used to extract reads belonging to each sample, which mapped in the genomic interval corresponding to exon 7 of the csd gene, harboring the HVR (Hav3.1_NC_037640.1:11771976-11772119) [10]. Sample-specific fasta files containing all the sequences falling within the HVR were created, and the CAP3 Sequence Assembly Program (−o 40, −p 90) [25] was used to cluster sequences and generate contigs. The CAP3 output was filtered, and only samples assembled in 2 contigs and less than two singletons were kept. As no sequencing depth cut-off was imposed prior to the assembly procedure, manual inspection was performed on all assemblies to verify and validate the performance of the procedure and discard poorly supported assemblies. The two contigs sequences retrieved for each sample, supposed to represent the sequences of the two alleles, were translated into all six frames with EMBOSS transeq (https://www.ebi.ac.uk/Tools/st/emboss_transeq/, accessed on 28 April 2021), and the two in-frame translations matching the csd amino acid sequences were retrieved [10,13,15]. In-frame translations resulting in truncated polypeptides were discarded. Putative HVR amino acid allele sequences obtained for all samples where de novo allele reconstruction was successful were aligned with Clustal Omega [26]. The HVR sequence from *Apis cerana* (GenBank QKY64514.1) was added to the dataset to be used for tree rooting. PhyML 3.0 [27] was used to produce a phylogenetic tree using the CpREV model of amino acid substitution. ITOL [28] was used to display the tree.

CAP3-derived putative HVR amino acid allele sequences were used to blast the *Apis mellifera* NCBI nr database and retrieve csd HVR known sequences through tBLASTn (amino acid query versus translated database). Only identical hits (100% coverage and 100% similarity) were retained.

## 3. Results

### 3.1. Csd HVR Allele Reconstruction and Classification

The pipeline aiming at the reconstruction of *csd* alleles using genomic resequencing data was applied to sequences from 125 honeybee diploid worker bees. In detail, quality-trimmed reads were mapped to the Amel_Hav3.1 honeybee genome, and 5310 mapped reads falling within the HVR genome coordinates (Hav3.1_NC_037640.1:11771976-11772119) were extracted and split according to the sample of origin. Sample-specific CAP3 generated contigs were inspected, and only sequences from the 87 samples for which the assembly procedure resulted in two contigs and not more than one singleton were retained. The two contigs sequences retrieved for each sample, supposed to represent the sequences of the two alleles, were translated into proteins. One hundred seventy-four sequences gained from 87 diploid samples were then filtered to 154 sequences due to 20 polypeptide sequences being truncated. Thus, among these 154 sequences, only those belonging to the 69 samples showing both alleles successfully reconstructed were kept, leading to 138 sequences.

Among the 138 reconstructed sequences, we found 88 different alleles, 32 were shared in more than one sample, while 56 were unique in our pool of sequences. These 88 sequences, which encompass all the *csd* alleles represented in our colonies collection that were successfully reconstructed, are reported along with their sequence length in Table 1. Among the 88 alleles, 68 were found in the NCBI GenBank database by BLAST analysis, leading to the identification of 20 novel *csd* alleles (Supplementary File S1). Further, all the sequences retrieved from GenBank showing 100% identity to our 68 alleles are given in Table 1.

### 3.2. Phylogenetic Tree

**Figure 1:**
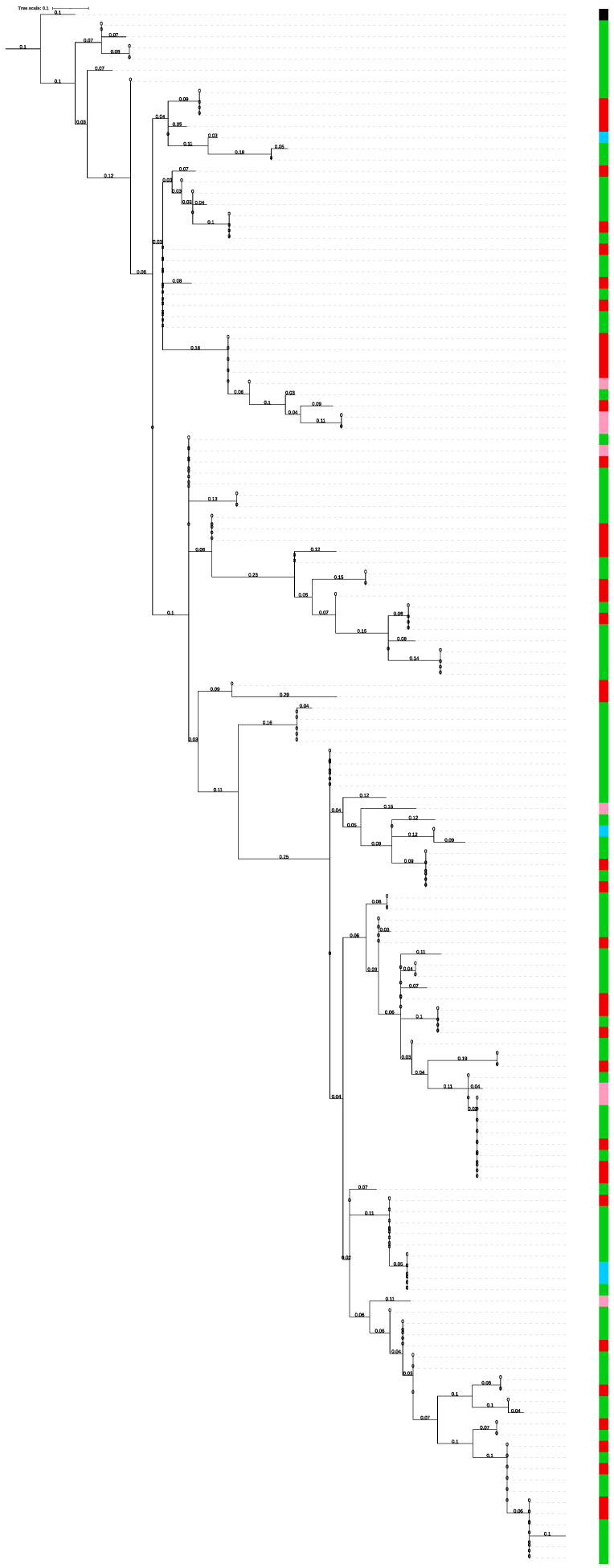
Phylogenetic tree based on the amino acid sequence of the *csd* alleles HVR region in 7 different Italian honeybee subspecies. Colors are given according to evolutionary lineages: *A. m. sicula* belongs to the African type (pink); *A. m. mellifera* belongs to the western and northern Europe lineage (blue); *A. m. carnica, A. m. cecropia* and *A. m. ligustica* belong to the eastern Europe group (green); Buckfast and hybrid Carnica are crosses of European subspecies belonging to C type (red). The tree was rooted on *Apis cerana* (black).

A phylogenetic tree was constructed based on the 138 *csd* HVR amino acid sequences retrieved from the seven *A. mellifera* subspecies represented in our collection (Figure 1). The seven subspecies are from three different evolutionary lineages: in detail, *A. m. sicula* belongs to the African type (A); *A. m. mellifera* belongs to the western and northern Europe lineage (M); *A. m. carnica*, *A. m. cecropia* and *A. m. ligustica* belong to the eastern Europe group (C); Buckfast and hybrid Carnica are crosses of European subspecies belonging to C type (EC) [29]. *A. cerana* was used to root the tree. The honeybee samples sub-grouping does not strictly reflect the evolutionary lineages.

### 3.3. Frequency Analysis

**Figure 2:**
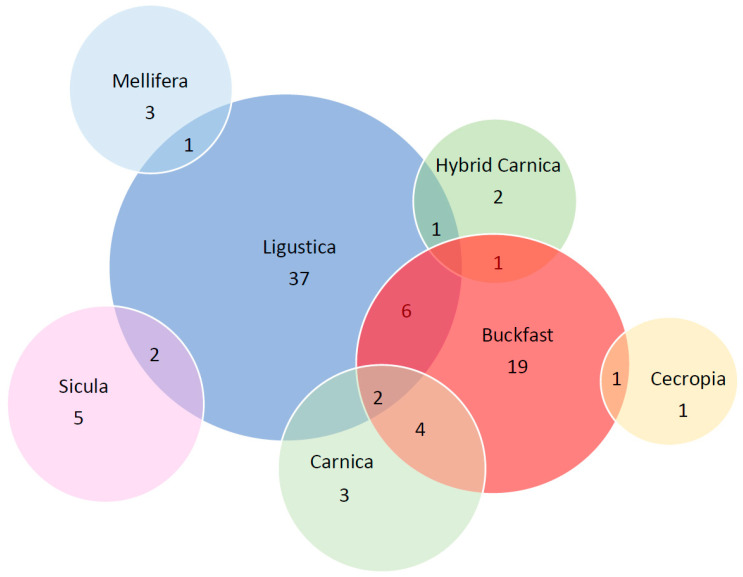
Venn diagram of the *csd* alleles among the 7 honeybee subspecies.

Figure 2 shows the Venn diagram of the allele distribution among subspecies. Of the 88 alleles (numbered from Allele 1 to Allele 88) reconstructed in our study, 70 are private alleles; 16 alleles are shared in two subspecies (2, 4, 7, 8, 9, 17, 18, 19, 21, 22, 24, 26, 27, 28, 29, 31), while 2 alleles (13, 16) are present in three subspecies (*A. m. ligustica*, *A. m. carnica* and Buckfast). 

Allele frequencies of alleles were computed and reported in Table 1. In detail, the most frequent allele in the entire population (allele 9, frequency 3.62%) is carried by five bees: four Buckfast and one *A. m. ligustica*.

The most frequent alleles within each subspecies were further explored:

- Among the 68 sequences belonging to 34 *A. m. ligustica* specimens, we identified 49 different alleles. Among these, alleles 1, 11 and 32 were the most frequent in *A. m. ligustica*.
- Among the 42 sequences belonging to 21 Buckfast individuals, we identified 33 different alleles. Allele 9 was the most frequent, with a frequency of 9.52%.
- Among the eight sequences belonging to four *A. m. sicula,* seven different alleles were identified; allele 30 was the most frequent, with a frequency of 25%,
- Among the 10 sequences belonging to five *A. m. carnica*, we identified nine different alleles with a frequency of 20%; allele 25 was the most frequent.
- The remaining three subspecies (hybrid Carnica, *A. m. mellifera*, *A. m. cecropia*) had a limited number of reconstructed *csd* HVR sequences (4, 4 and 2, respectively), each bearing a different allele.

## 4. Discussion

A reduction of the number of *csd* alleles in the population and, consequently, an increase in colony losses can cause problems to honeybee biodiversity conservation strategies as well as in honeybee-managed colonies. It is therefore essential to analyze, quantify and conserve the highest number and diversity of *csd* alleles in the honeybee populations worldwide. 

In the first studies, csd alleles were determined by Sanger sequencing based on haploid drone samples [19,20] or by high-resolution melting analysis [15]. The first study to use high-throughput sequencing focusing on this locus showed an alternate method to Sanger sequencing: sample-specific amplified *csd* alleles were Illumina sequenced, clustered, compared to the reference honeybee *csd* sequence, filtered and translated to yield a discrete number of alleles [17]. The results, based on deep sequencing of the region under analysis, were supported by GenBank data gained using Sanger technology showing 100% identity. A recent study used Ion Torrent on honey samples to extract, trim and cluster reads, which were then translated to obtain *csd* alleles [18].

Our focus was to study *csd* variability by analyzing existing next-generation resequencing data through a semi-automated pipeline to reconstruct both *csd* alleles from diploid honey bees. The pipeline encompasses manual curation steps, which are essential due to the nature of the available data, which were produced with different aims and did not provide a sequencing depth compliant with conventional de novo assembly procedures. Nonetheless, in many situations, curated reference-independent sequence assembly was possible. In our study, *csd* HVR allele reconstruction was obtained for 70.4% of the total samples in our collection. Our results are supported by comparison to known alleles present in GenBank, showing 100% identity of 68 out of 88 alleles, the 20 remaining alleles representing variability at the *csd* HVR locus explored for the first time. 

The 88 different *csd* alleles identified among the 138 reconstructed amino acid sequences belong to seven different honeybee subspecies; this number of alleles is in agreement with previous studies on large collections of bees, in which a large number of alleles were observed [9,19,20,21]. If we consider the ratio between the observed alleles and the total number of sequences analyzed, our result of 0.63 is comparable with other studies that report results on smaller sample sets. Cho et al. identified 18 distinct alleles, sampling 27 workers from a single hive; for this reason, they hypothesized that the total number of circulating *csd* alleles should be very high [13]. In 2019, Kaskinova et al. analyzed 15 different colonies (sampling 2-3 haploid drones per colony), finding 20 different HVR sequences with a ratio of 0.66 (20/30), which is comparable with our results [16]. Moreover, the 88 alleles found in our study differ in amino acid sequence and length. As shown in Table 1, the length varies from 27 to 50 amino acids, in agreement with Kaskinova et al. [16].

The phylogenetic tree (Figure 1) generated from all the reconstructed sequences at our disposal from the seven subspecies belonging to three different evolutionary lineages shows mixed distribution of the subspecies, forming clades that do not reflect the evolutionary lineages. Our result is similar to the phylogenetic tree constructed by Wang et al. on six different subspecies (*A. m. anatolica, A. m. caucasica, A. m. carnica, A. m. carpatica, A. m. ssp.* and *A. m. ligustica*) belonging to two different geographical types [19]. This result might be related to the high frequency of mutations of this locus that continuously create new variability [20].

However, it is interesting to note and observe from the Venn diagram that two alleles (alleles 13 and 16) are shared between three different subspecies *A. m. ligustica, A. m. carnica* and Buckfast. It is known that the three subspecies belong to the same evolutionary lineage C type (Figure 2) and often share the same geographical area. However, a phylogenetic tree based on a whole genome SNP dataset of the same sample set, excluding the *A. m. cecropia* sample [24], identified a distinctive subgrouping of samples based on their subspecies. Furthermore, as already stated by Kaskinova et al. [16], no correspondence was observed between the structure of the phylogenetic tree of *csd* haplotypes and the structure of the phylogenetic tree of honeybee subspecies obtained on the basis of the analysis of microsatellite loci and mitochondrial DNA. Our results further support the observation that this specific region, although being very variable, does not allow for discriminating among honeybee subspecies and consequently is not suitable for inferring phylogenetic relationships at the population level [14].

Furthermore, our results confirm the uneven distribution of *csd* alleles in *Apis mellifera* populations with many alleles present in a single bee (infrequent alleles) and other alleles that show a high frequency [20]. In detail, Allele 9 was identified in our study as the most frequent allele present in five samples belonging to two different subspecies. The same allele was identified in more than one sample in a consolidated report on *csd* alleles by Bilodeau et al. in 2021 (named Allele Amel Csd-HVR57 in their report) [21].

## 5. Conclusions

In conclusion, we used existing 150 bp Illumina whole genome resequencing data to explore the hypervariable region (HVR) of the *csd* gene of 125 diploid worker bees and investigated allelic variability in the complementary sex determiner gene (*csd*). We identified 88 different alleles, 68 of which match with *csd* alleles already present in the NCBI GenBank database, while 20 are newly described alleles. Given that the viability of the brood requires a high number of circulating alleles, high allelic variation should be a priority in both subspecies as well as in highly selected honeybee breeding lines. A representation of circulating alleles can be obtained by both traditional Sanger and NGS-based sequencing approaches. To this end, NGS-based *csd* assessment offers the availability of a method to rapidly generate *csd* alleles population information, starting from whole genome data created for selection. This information can enable beekeepers to monitor *csd* variability in selective breeding programs and to perform traditional selection at the same time. This solution should be actively promoted among bee-breeders.

## Figures and Tables

**Table 1:** Amino acid sequences of alleles belonging to the hypervariable region of the *csd* gene, their lengths (L = nr of aa) and frequencies. If the allele was identified in GenBank, the accession numbers are shown (GenBank ID). In bold are the novel alleles identified in the study.

Allele ID	Amino Acid Sequences	L	Freq. in the Population	Freq. in the Subspecies	GenBank ID
**Allele 1**	**SSLSNKTIHNNNNYKYNYNNNNYNNNNYNNNYNNNCKKLYYNINYIEQI**	**49**	**2.17%**	**Ligustica: 4.41%**	
Allele 2	SSLSNNYNYNNYNNNYKPLYYNINYIEQI	29	2.17%	Ligustica: 2.94%	AAS86659.1, AAS86660.1, AAS86661.1, AAS86663.1, ABD14105.1, ABD14106.1, ABD14107.1, ABD14108.1, AGA84527.1
				Buckfast: 2.38%	
Allele 3	SSLSNNYNSNSYNNYNNNYKKLQYYNIINIEQI	33	1.45%	Ligustica: 2.94%	CCF23480.1, QEN96041.1
**Allele 4**	**SSLSNNTIHNNNYKYNYNNNYNNYNNYKKLYYNINYIEQI**	**40**	**1.45%**	**Ligustica: 1.47%**	
				**Buckfast: 2.38%**	
Allele 5	SSLSNKTIHNNNNYKYNYNNNYNNNNNYSKKLYYNINYIEQI	42	2.17%	Ligustica: 4.41%	AQZ41186.1, AQZ41196.1, AQZ41212.1, QEN96050.1
Allele 6	SSLSNNYNYSNYNNYNNYNNNYNNYKKLYYNINYIEQI	38	1.45%	Ligustica: 2.94%	CCF23508.1
Allele 7	SSLSKNTIHNNNYKYNYNNNNNYNNNYKKLQYYNINYIEQI	41	2.90%	Ligustica: 2.94%	CCF23518.1
				Buckfast: 4.76%	
Allele 8	SSLSNSCNYSNNYYNKKLYYNIINIEQI	28	2.17%	Ligustica: 2.94%	CCF23469.1
				Buckfast: 2.38%	
Allele 9	SSLSNKTIHNNNNYKYNYNNKYNYNNNNYNKKLYYKNYIINIEQI	45	3.62%	Ligustica: 1.47%	ABD14097.1, QEN96103.1
				Buckfast: 9.52%	
Allele 10	SSLSNNYNYSNYNNYNNNYNNYKKLYYNINYIEQI	35	1.45%	Ligustica: 2.94%	ABV56215.1
Allele 11	SSLSNNYISNISNYNNNNNSKKLYYNINYIEQI	33	2.17%	Ligustica: 4.41%	AGZ61876.1, CCF23481.1, CCF23482.1
Allele 12	SSLSKNTIHNNNYKYNYNNNNYNNSKKLYYNINYIEQI	38	1.45%	Ligustica: 2.94%	CCF23507.1
				Ligustica: 1.47%	
Allele 13	SSLSNKTIHNNNNYKYNYNNNNYKNYNNYKKLYYNINYIEQI	42	2.17%	Carnica: 10.00%	AQZ41220.1
				Buckfast: 2.38%	
**Allele 14**	**SSLSNKTIHNNNNYNNYKKLYYNIINIEQI**	**30**	**1.45%**	**Ligustica: 2.94%**	
Allele 15	SSLSNNTIHNNNYKYNNYNNYNKKLYYNIINIEQI	35	1.45%	Ligustica: 2.94%	CCF23492.1
				**Carnica: 10.00%**	
**Allele 16**	**SSLSNKTIHNNNNYNNNNYNNYKKLYYNINYIEQI**	**35**	**2.17%**	**Buckfast: 2.38%**	
				**Ligustica: 1.47%**	
Allele 17	SSLSNNTIHNNNNYNKKLYYNIINIEQI	28	1.45%	Ligustica: 1.47%	AGZ61866.1, AGZ61871.1, AGZ61872.1, AGZ61874.1, CCF23470.1
				Buckfast: 2.38%	
Allele 18	SSLSNKTIHNNNNYKYNYNNNCKKLYYNINYIEQI	35	1.45%	Hyb. Carn: 25.00%	ABD14096.1, QEN96024.1, QEN96028.1
				Ligustica: 1.47%	
Allele 19	SSLSNNYKYSNYNNYNNNYNNNYNNNYNNNYKKLYKNYIINIEQI	45	2.90%	Hyb. Carn: 25.00%	ABD14145.1, ABD14146.1, ADJ57940.1
				Buckfast: 7.14%	
Allele 20	SSLSNNYNSNNYNKYNYNNSKKLYYNINYIEQI	33	2.17%	Buckfast: 7.14%	CCF23483.1
Allele 21	SSLSNKTIHNNNNYNNNNYNNYKKLYYNIINIEQI	35	1.45%	Carnica: 10.00%	AEI99777.1
				Buckfast: 2.38%	
Allele 22	SSLSNNYKYSNYNNYNNNYNNYNNNYNNNYKKLYYNINYIEQI	43	1.45%	Carnica: 10.00%	CCF23533.1
				Buckfast: 2.38%	
Allele 23	SSLSNHYNYNNNKYNNYNNDYKKLYYNINYIEQI	34	1.45%	Ligustica: 2.94%	ADJ57943.1, AQZ41194.1, AQZ41195.1
Allele 24	SSLSNKTIHNNNNYKYNYKNYNNSKKLYYNVINIEQI	37	1.45%	Buckfast: 2.38%	AEI99780.1, AEI99781.1, AEI99790.1
				Cecropia: 50.00%	
Allele 25	SSLSNKTIHNNNNYNNYKKLYYNINYIEQI	30	1.45%	Carnica: 20.00%	AQZ41206.1, AQZ41207.1, AQZ41208.1, CCF23474.1
Allele 26	SSLSNKTIHNNNKYNYNKYNYNNNNYNNYKKLYYNINYIEQI	42	1.45%	Carnica: 10.00%	CCF23526.1
				Buckfast: 2.38%	
Allele 27	SSLSNNYNYNNNNYNNYNNNYNNNYNKKLYYNIINIEQI	39	2.17%	Carnica: 10.00%	AQZ41204.1, AQZ41205.1, CCF23512.1
				Buckfast: 4.76%	
Allele 28	SSLSNKTIHNNNNYKYNYNNNNYNNNYNNNCKKLYYNIINIEQI	44	1.45%	Mellifera: 25.00%	AEI99762.1, QEN96035.1
				Ligustica: 1.47%	
Allele 29	SSLSNNYNYNNNNYNNNYNKKLYYNINYIEQI	32	1.45%	Ligustica: 1.47%	ADJ57960.1, AEI99714.1, AEI99724.1, AEI99729.1, AEI99733.1, AEI99735.1, AEI99736.1, AEI99738.1, AEI99741.1, AEI99743.1, AEI99744.1, AEI99755.1, AEI99759.1
				Sicula: 12.50%	
**Allele 30**	**SSLSNNYNSNNYYNYNNNKKLYYKNYIINIEQI**	**33**	**1.45%**	**Sicula: 25.00%**	
Allele 31	SSLSNKTIHNNNNYKYNYNNKYNYNNNNYNNNNYNKKLYYKNYIINIEQI	50	2.17%	Sicula: 12.50%	AQZ41179.1, AQZ41181.1, AQZ41192.1, AQZ41193.1, QEN96025.1
				Ligustica: 2.94%	
Allele 32	SSLSNSCNYSNNYNNNYNNTKKLYYNINYIEQI	33	0.72%	Ligustica: 4.41%	AEI99783.1, AEI99788.1, AEI99792.1, AGZ61869.1
Allele 33	SSLSNKTIHNNNNYKNYNYKKLYYNIINIEQI	32	0.72%	Ligustica: 1.47%	CCF23479.1
Allele 34	SSLSNNYNYSNYNNNNYKQLCYNINYIEQI	30	0.72%	Ligustica: 1.47%	ABD14139.1, ABD14141.1, ABD14142.1, ABD14143.1, ABD14144.1, AGA84531.1, AGZ61875.1, QEN96094.1
Allele 35	SSLSNNYNYSNYNNYNNYNNNYNNYNNNYNNYKKLYYNINYIEQI	45	0.72%	Buckfast: 2.38%	CCF23536.1
Allele 36	SSLSNNYNSNSYNNYNNNYYNNKKLQYYNINYIEQI	36	0.72%	Ligustica: 1.47%	AGA84533.1, CCF23490.1, QEN96078.1
Allele 37	SSLSNKTIHNNNNYNNNNYNNYNNNNYNNYKKLYYNIINIEQI	43	0.72%	Buckfast: 2.38%	QEN96087.1
Allele 38	SSLSSNYNSNNYNNYNNYKQLCYNINYIEQI	31	0.72%	Mellifera: 25.00%	ART88596.1, CCF23477.1
Allele 39	SSLSNNYNYNNNKYNYNNNNYKQLCYNINYIEQI	34	0.72%	Ligustica: 1.47%	AEI99717.1, AEI99718.1, AEI99720.1, AEI99721.1, AEI99726.1, AEI99732.1, AEI99734.1, AEI99740.1, AEI99742.1, AGA84529.1
Allele 40	SSLSNKTIHNNNNYNNNNYNNYNNNNYNNYKKLYYNINYIEQI	43	0.72%	Ligustica: 1.47%	CCF23531.1
Allele 41	SSLSNNYKYSNYNNYNNNNYNNNNYNNNSKKLYYNIINIEQI	42	0.72%	Ligustica: 1.47%	AGA84526.1, CCF23524.1
Allele 42	SSLSNKTIHNNNNYNNNNYKKLQYYNINYIEQI	33	0.72%	Ligustica: 1.47%	ABV56220.1, AQZ41221.1, QEN96101.1
Allele 43	SSLSNNYNYNNNNYNNYNNNYNNNYNKKLYYNINYIEQI	39	0.72%	Ligustica: 1.47%	QEN96052.1, QEN96059.1
**Allele 44**	**SSLSNKTIHNNNYKYNYYNNNNYKKLQYYNIINIEQI**	**37**	**0.72%**	**Ligustica: 1.47%**	
Allele 45	SSLSNNYNYNNNNYNNYNNYNNYNNNYNKKLYYNINYIEQI	41	0.72%	Ligustica: 1.47%	ABV56219.1
Allele 46	SSLSNNYKYSNYNNYNNYNKKLYYKNYIINIEQI	34	0.72%	Ligustica: 1.47%	ABD14104.1, ADJ57958.1, AGA84523.1, QEN96085.1, QEN96088.1
**Allele 47**	**SSLSNNYNYNNNNYNNYNNYNNNYNNNYNKKLYYNINYIEQI**	**42**	**0.72%**	**Ligustica: 1.47%**	
Allele 48	SSLSNNYKYSNYNNNNYNNNSKKLYYNINYIEQI	34	0.72%	Ligustica: 1.47%	AQZ41199.1, CCF23485.1
**Allele 49**	**SSLSNKTIHNNNNYNNNNYNKKLYYNINYIEQI**	**33**	**0.72%**	**Ligustica: 1.47%**	
Allele 50	SSLSNKTIHNNNNYKYNYNNNNYNNNYNNYKKLYYNINYIEQI	43	0.72%	Ligustica: 1.47%	CCF23532.1, QEN96032.1, QEN96033.1, QEN96042.1
Allele 51	SSLSNKTIHNNNNYKNYNNYKNYNNYKKLYYNINYIEQI	39	0.72%	Carnica: 10.00%	CCF23513.1
Allele 52	SSLSNKTIHNNNNYKYNYNNNNYNNNNYNKKLYYKNYIINIEQI	44	0.72%	Ligustica: 1.47%	CCF23527.1
Allele 53	SSLSNNTIHNNNYKYNYNNKYNYNNKKLYYNIINIEQI	38	0.72%	Carnica: 10.00%	CCF23510.1, QEN96012.1
Allele 54	SSLSNKTIHNNNNYKYNYNNNNYNNNNYNNNYNNNCKKLYYNIINIEQI	49	0.72%	Buckfast: 2.38%	AAQ57659.1, QEN96069.1
Allele 55	SSLSNNYNSNSYNNNYNNNYYNKKLQYYNINYIEQI	36	0.72%	Buckfast: 2.38%	CCF23486.1
**Allele 56**	**SSLSNNYKYSNYNNYNNYNNNNYNNYNNYNNKKLYYNIINIEQI**	**44**	**0.72%**	**Buckfast: 2.38%**	
Allele 57	SSLSNKTIHNNNNYKKLYYNINYIEQI	27	0.72%	Buckfast: 2.38%	CCF23466.1
Allele 58	SSLSNNYNYSNYNNYNNYKKLYYNINYIEQI	31	0.72%	Buckfast: 2.38%	CCF23475.1
Allele 59	SSLSNNTIHNNNYKYNYNNNNYNNNNYNKKLYYNIINIEQI	41	0.72%	Buckfast: 2.38%	ABD14102.1, ABD14103.1, AEI99760.1, AEI99784.1
Allele 60	SSLSNNYNYSNYNNYNNNNNYNNYKKLYYNINYIEQI	37	0.72%	Ligustica: 1.47%	ABD14109.1, ABD14110.1, ABD14111.1, ABD14112.1, ABD14113.1, ABD14114.1, ABD14115.1, ABD14116.1, AQZ41223.1, QEN96102.1
Allele 61	SSLSNNYKYSNYNNYNNNYNNYNNNYKKLYYNINYIEQI	39	0.72%	Ligustica: 1.47%	AEI99754.1, AEI99786.1, AEI99787.1, AEI99789.1
Allele 62	SSLSSSCNYSNNYNNYYNNNKKLYYNIINIEQI	33	0.72%	Ligustica: 1.47%	AGA84525.1, AIS73042.1, AIS73050.1, CCF23484.1, QEN96007.1, QEN96009.1, QEN96018.1, QEN96019.1, QEN96026.1, QEN96061.1, QEN96074.1, QEN96084.1, QEN96097.1, QEN96105.1
**Allele 63**	**SSLSNKTIHNNNKYNYNNNYNNNCKKLYYNINYIEQI**	**37**	**0.72%**	**Cecropia: 50.00%**	
Allele 64	SSLSNNRNSNNYNNYNYKKLYYNINYIEQI	30	0.72%	Buckfast: 2.38%	ABV56222.1, CCF23473.1
Allele 65	SSLSNNYNYSNYNNYNNNYNNNYNNNDYKKLYYKNYIINIEQI	43	0.72%	Buckfast: 2.38%	ABV56216.1, QEN96106.1
Allele 66	SSLSNNYNYSNYNNYNNNNYNNYKKLYYNINYIEQI	36	0.72%	Hyb. Carn: 25.00%	CCF23496.1
Allele 67	SSLSNNYNYSNNYNNYYNNNNNYNNYKKLYYNIINIEQI	39	0.72%	Hyb. Carn: 25.00%	AEI99727.1, AEI99728.1
Allele 68	SSLSKNTIHNNNYNNSKKLYYNIINIEQI	29	0.72%	Buckfast: 2.38%	AEI99716.1
**Allele 69**	**SSLSNKTIHNNNNYNNNYNNNCKKLYYNIINIEQI**	**35**	**0.72%**	**Mellifera: 25.00%**	
**Allele 70**	**SSLSNKTIHNNNNYNNNNYNNNNYNNNNYKKLQYYNINYIEQI**	**43**	**0.72%**	**Mellifera: 25.00%**	
**Allele 71**	**SSLSNNYKYSNYNNYNNNNYKKLQYYNINYIEQI**	**34**	**0.72%**	**Ligustica: 1.47%**	
Allele 72	SSLSNKTIHNNNNYKYNYNNNNYKPYYNINYIEQI	35	0.72%	Ligustica: 1.47%	AEI99745.1
**Allele 73**	**SSLSNKTIHNNNNYKYNYNNNYKKLYYKNYIINIEQI**	**37**	**0.72%**	**Sicula: 12.50%**	
**Allele 74**	**SSLSNKTIHNNNNYKYNYNNNYNNNSKKLQYYYNINYIEQI**	**41**	**0.72%**	**Sicula: 12.50%**	
**Allele 75**	**SSLSNKTIHNNNYKYNYNNKHNYNKLYYNINYIEQI**	**36**	**0.72%**	**Sicula: 12.50%**	
Allele 76	SSLSNNYKYSNYNNYNNYNNNSKKLYKNYIINIEQI	36	0.72%	Sicula: 12.50%	AQZ41187.1, AQZ41197.1, AQZ41198.1, ART88598.1, CCF23491.1, QEN96020.1, QEN96040.1
Allele 77	SSLSNKTIHNNNNYNNNNYNNYNNNNYNYKKLYYNINYIEQI	42	0.72%	Ligustica: 1.47%	CCF23528.1
Allele 78	SSLSNKTIHNNNNYKYNYNNNYNNNSKKLYYNINYIEQI	39	0.72%	Ligustica: 1.47%	AGA84537.1, CCF23514.1
Allele 79	SSLSNNYNYSNYNNYNNNYNNYNKKLYYNINYIEQI	36	0.72%	Buckfast: 2.38%	ABD14117.1
**Allele 80**	**SSLSNKTIHNNNNYKNYNNYKNYNNYKNYNNYKKLYYNINYIEQI**	**45**	**0.72%**	**Buckfast: 2.38%**	
Allele 81	SSLSNNYNYNNYNNTNNINKQLYYNINYIEQI	32	0.72%	Buckfast: 2.38%	ABV56218.1, AQZ41218.1, QEN96077.1, QEN96095.1
Allele 82	SSLSNNYSYNNYNNNNYNKKLYYNINYIEQI	31	0.72%	Buckfast: 2.38%	CCF23476.1, QEN96068.1
Allele 83	SSLSNNYNYNNNNYNNYNNNYNKKLYYNINYIEQI	35	0.72%	Buckfast: 2.38%	AQZ41215.1, AQZ41216.1, AQZ41217.1, CCF23487.1
**Allele 84**	**SSLSNNYNYSNYNNYNNNNNYNNNNYNYKKLYYNINYIEQI**	**41**	**0.72%**	**Buckfast: 2.38%**	
**Allele 85**	**SSLSNKTIHNNNNNYNNYNKKLYYNIINIEQI**	**32**	**0.72%**	**Ligustica: 1.47%**	
Allele 86	SSLSNNTIHNNNNYKYNYNNNYNNYNNYNNKKLYYNIINIEQI	43	0.72%	Buckfast: 2.38%	CCF23529.1
**Allele 87**	**SSLSTNTIHNNNNYKYNYNNNYNNYNNKKLYYNINYIEQI**	**40**	**0.72%**	**Ligustica: 1.47%**	
Allele 88	SSLSNNYISNISNYNNNNNYNKKLYYNINYIEQI	34	0.72%	Ligustica: 1.47%	AAQ67418.1, ABD14119.1, DAA06292.1, QEN96014.1, QEN96029.1, QEN96031.1, QEN96036.1, QEN96038.1, QEN96045.1, QEN96055.1, QEN96057.1, QEN96079.1

## Data Availability

Amino acid sequences of the *csd* alleles are given in the manuscript. Nucleotide sequences of the novel *csd* hypervariable regions described in this work are available in fasta format (Supplementary File S1), while raw genome data and phenotypes are part of a reference population used for selection by commercial breeders and have commercial value. Therefore, restrictions apply to the availability of these data, which are not publicly available. The authors can be contacted for a specific request.

## Supplementary Materials

The following supporting information can be downloaded at: https://www.mdpi.com/article/10.3390/genes13060991/s1, Supplementary File S1: Nucleotide sequences of the novel *csd* hypervariable regions.
